# Protein Considerations for Optimising Skeletal Muscle Mass in Healthy Young and Older Adults

**DOI:** 10.3390/nu8040181

**Published:** 2016-03-23

**Authors:** Oliver C. Witard, Sophie L. Wardle, Lindsay S. Macnaughton, Adrian B. Hodgson, Kevin D. Tipton

**Affiliations:** 1 Health & Exercise Sciences Research Group, Faculty of Health Sciences and Sport, University of Stirling, Stirling FK9 4LA, UK; 2 Suntory Beverage Food Europe, 2 Longwalk Road, Stockley Park, Uxbridge UB11 1BA, UK

**Keywords:** muscle hypertrophy, muscle protein synthesis, amino acid availability, protein source, protein dose, protein timing, protein pattern, macronutrient coingestion

## Abstract

Skeletal muscle is critical for human health. Protein feeding, alongside resistance exercise, is a potent stimulus for muscle protein synthesis (MPS) and is a key factor that regulates skeletal muscle mass (SMM). The main purpose of this narrative review was to evaluate the latest evidence for optimising the amino acid or protein source, dose, timing, pattern and macronutrient coingestion for increasing or preserving SMM in healthy young and healthy older adults. We used a systematic search strategy of PubMed and Web of Science to retrieve all articles related to this review objective. In summary, our findings support the notion that protein guidelines for increasing or preserving SMM are more complex than simply recommending a total daily amount of protein. Instead, multifactorial interactions between protein source, dose, timing, pattern and macronutrient coingestion, alongside exercise, influence the stimulation of MPS, and thus should be considered in the context of protein recommendations for regulating SMM. To conclude, on the basis of currently available scientific literature, protein recommendations for optimising SMM should be tailored to the population or context of interest, with consideration given to age and resting/post resistance exercise conditions.

## 1. Introduction

Skeletal muscle is crucial for metabolic health and sport performance. Beyond the positive relationship between skeletal muscle mass (SMM), strength and athletic performance, skeletal muscle also plays an important, and often underappreciated, role in reducing risk of diseases such as obesity, cardiovascular disease, insulin resistance, diabetes and osteoporosis [1]. Therefore, strategies to preserve or increase SMM are vitally important for both clinical and athletic populations.

Skeletal muscle tissue displays remarkable plasticity. This plasticity allows for adaptation, including an increase in SMM. Skeletal muscle proteins are continuously being remodelled through the simultaneous processes of muscle protein synthesis (MPS) and muscle protein breakdown (MPB). In turn, skeletal muscle protein remodeling is a prerequisite for increasing SMM [2]. Exercise and nutrition influence SMM through changes in MPS more than MPB [3]. Thus, MPS is accepted to be the dominant process of muscle remodelling responsible for regulating SMM in healthy adult humans. Whilst a high degree of muscle remodelling also is associated with other phenotypic adaptations, including the repair of old and/or damaged muscle proteins and modifications to the type and functionality of muscle proteins, the present review refers to skeletal muscle protein remodelling in the context of optimising muscle mass.

**Figure 1:**
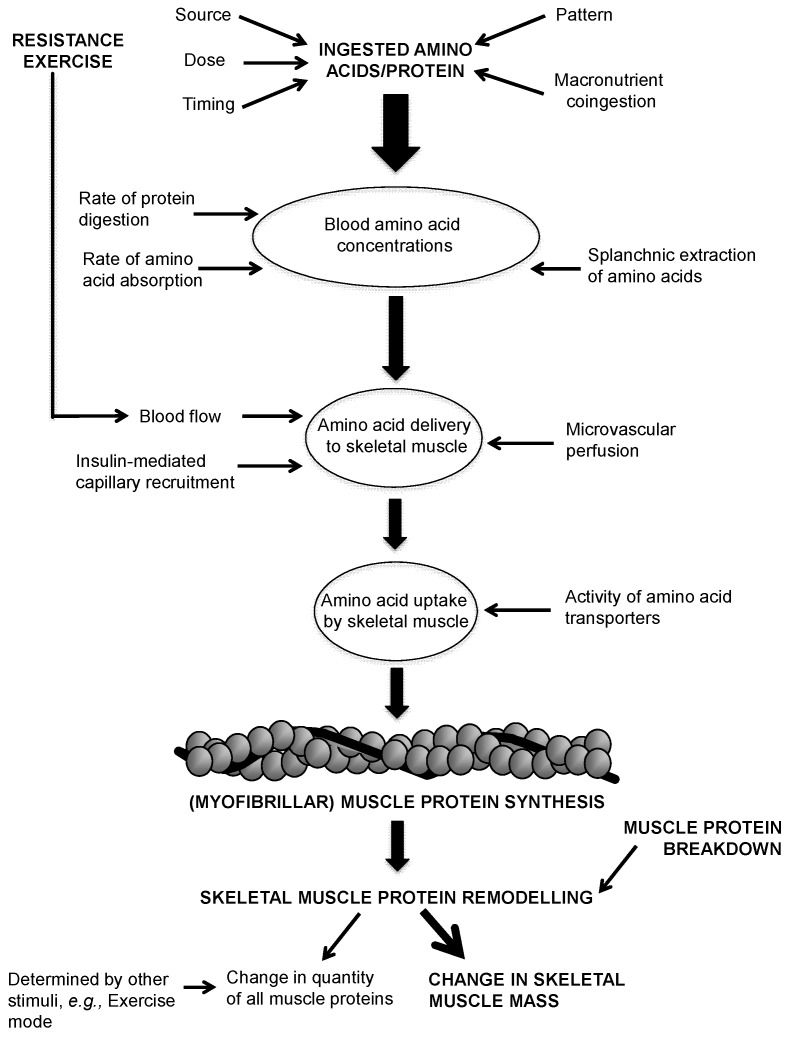
Simplified diagram detailing the role of amino acid availability in regulating muscle protein synthesis with amino acid/protein ingestion and exercise. Whilst resistance exercise preferentially stimulates the synthesis of contractile myofibrillar proteins (e.g., actin, myosin, troponin), resistance exercise also stimulates the synthesis of non-contractile proteins (e.g., mitochondrial and sarcoplasmic) in skeletal muscle.

Protein or amino acid feeding stimulates MPS at rest [4] and during exercise recovery [5]. Thus, it follows that protein ingestion is a key stimulus for preserving SMM under resting conditions and increasing SMM under exercise training conditions. The stimulation of MPS is fundamentally regulated by extracellular and intracellular amino acid availability [6]. Figure 1 depicts the role of amino acid availability in regulating MPS in response to amino acid/protein ingestion and exercise. Amino acid availability is modulated by several dietary factors, including the amino acid/protein source, amount ingested (as a single dose), timing, pattern and macronutrient coingestion. These factors independently and synergistically impact rates of protein digestion and amino acid absorption, the splanchnic extraction of amino acids, microvascular perfusion (capillary recruitment and dilation), the delivery of amino acids to skeletal muscle and the uptake of amino acids by skeletal muscle, and thus regulate postprandial rates of MPS. In addition, exercise enhances the ability of skeletal muscle to respond to amino acid provision [7,8]. The most likely contributing mechanism is an exercise-induced increase in blood flow to the muscle [5] that increases the delivery of amino acids to the muscle, thus increasing the provision of substrate for MPS [9]. Crucially, the responsiveness of MPS to amino acid ingestion deteriorates with advancing age [10,11,12]. This phenomenon is referred to as “anabolic resistance” and is thought to be mediated by impairments in each of the dietary factors introduced above.

To our knowledge, no previous authors have conducted a narrative review, using a systematic search strategy, to evaluate scientific evidence used to inform the latest protein recommendations for optimising MPS and SMM in healthy adult humans. Therefore, the primary objective of this review was to examine the impact of five key factors related to protein nutrition that regulate MPS, defined herein as:

- Amino acid/protein *source* refers to the origin source of ingested protein, e.g., isolated intact whey, casein or soy; animal or plant. Amino acid/protein *form* refers to the matrix form of ingested protein, e.g., liquid or solid.
- Amino acid/protein *dose* refers to the quantity of amino acids/protein contained in a single serving.
- Amino acid/protein *timing* refers to the timed intake of amino acids/protein in relation to exercise (before and after) or to ingestion of other nutrients.
- Amino acid/protein *pattern* refers to the distribution pattern of ingested amino acids/protein over a given period of time, accounting for the dose, timing and frequency of Amino acid/protein ingestion.
- *Macronutrient co-ingestion* refers to the concurrent ingestion of carbohydrate (CHO) and/or fat alongside an amino acid/protein source.

For clarity, this review has been structured to address each factor of protein nutrition independently. However, an important point of discussion concerns the interaction of these factors for modulating MPS in healthy young and older adults. An understanding of recommended protein nutrition practice for optimising MPS and SMM could lead to the provision of improved advice to aid the muscle health of young and older adults.

## 2. Methods

A systematic search strategy was employed to identify citations for this narrative review. We searched the National Library of Medicine database (PubMed) and Web of Science from their inception through to December 2015. The terms “muscle anabolism” OR “muscle protein synthesis” OR “muscle hypertrophy” OR “skeletal muscle protein remodelling” AND “protein feeding” OR “protein ingestion” OR “protein supplementation” OR “AA ingestion” AND “humans” were entered into both databases and filters including “articles” and “humans” were used to refine the search. After initial screening of title and abstracts, selected papers were examined, including the reference lists of the retrieved articles.

Studied participants met the eligibility criteria if classified as healthy with no medical contraindications. Participants were young (mean age of studied cohort ≤35 years) and older (mean age of studied cohort ≥65 years) adult men and women, resistance-trained (≥2 exercise sessions/week) or untrained volunteers, who were studied under resting or post resistance exercise conditions in the fed or fasted state. Several exclusion criteria were applied. We excluded intervention studies where the control condition was not considered appropriate to answer the question. For example, in the context of macronutrient coingestion, several studies included an iso-energetic CHO only [13] or a non-energetic placebo [14] rather than an amino acid/protein- only control condition. Additionally excluded were case studies and descriptive studies whereby no control group was used. Studies were excluded if they had a specific purpose of weight loss, if the method of protein intake was not oral (e.g., nasogastric/enteral intake of protein or the infusion of amino acids) and the exercise mode was not resistance-based. Finally, we excluded studies where participants were classified as patient groups (*i.e.*, not healthy, including overweight) and any non-human studies. Screening of studies resulted in the assessment of 64 citations for this narrative review. Of these, 24 citations were focused on amino acid/protein source, 8 dose, 11 timing, 6 pattern, and 15 macronutrient coingestion.

## 3. Synthesis of Findings

### 3.1. Amino Acid/Protein Source

Amino acid composition and digestive properties can vary between different isolated types of intact proteins, protein blends *vs.* isolated intact proteins and different forms of the same protein source. The Digestible Indispensable Amino Acid Score (DIAAS) is the latest and preferred index for differentiating between protein sources. The DIAAS score reflects the essential amino acid (EAA) content and digestion properties of any given protein source.

#### 3.1.1. Isolated Types of Intact Protein

The most common comparison of intact proteins is between rapidly digested whey protein that is high in leucine content (~12.5% of total protein) and slowly digested casein protein that exhibits a relatively lower (~8.5% of total protein) leucine content. Studies in young [15] and older [16,17] adults have consistently demonstrated a greater resting postprandial stimulation of mixed-MPS with ingestion of whey compared with casein protein. However, studies that compared the response of MPS or net muscle protein balance (NBAL; difference between MPS and MPB and thus indicative of the aggregate muscle protein anabolic response) to the post-exercise ingestion of whey and casein protein report equivocal results in both young [15,18,19] and older [16,20] adults. In young adults, studies report both a greater post-exercise response of mixed-MPS to ingestion of whey protein compared with micellar casein protein [15] and also no differences in the post-exercise response of NBAL (measured over 5 h) [19] and myofibrillar-MPS (measured over a 6 h period) [18] between whey and casein conditions. Additionally, a recent study in young adults reported no difference in the chronic resistance training-induced increase in lean body mass (LBM) between whey and casein protein conditions [21]. Similarly, studies in older adults have reported both a greater post-exercise stimulation of myofibrillar-MPS (measured over a 4 h period) following ingestion of whey protein isolate compared to micellar casein [16] and also no difference in the post-exercise response of mixed-MPS (measured over a 6 h period) [20] between whey and casein protein conditions. No longitudinal endpoint study in older adults has compared intact whey and casein protein sources on any outcome measure of SMM.

The discrepant findings between studies that fed whey and casein protein after exercise, at least in terms of acute measurements of MPS and NBAL, may be reconciled by general differences in study design. These differences include the form of intact protein ingested post-exercise (whey hydrolysate, whey isolate, micellar casein or calcium caseinate), the chosen endpoint measurement of muscle anabolism (e.g., mixed-MPS, myofibrillar-MPS or NBAL) and/or the time period over which MPS or NBAL was measured after protein ingestion. Micellar casein is insoluble and therefore is often treated with alkaline compounds such as calcium hydroxide to produce calcium caseinate. This treatment alters the digestion kinetics of casein, such that the rate of blood amino acid appearance with caseinate ingestion more closely mimics whey protein compared with micellar casein protein. Interestingly, acute studies that reported a differential post-exercise response of MPS between whey and casein protein ingestion administered micellar casein [15,16]. Conversely, those studies that reported a similar post-exercise response of MPS or NBAL between whey and casein protein conditions administered calcium caseinate protein [18,19,20]. Taken together, these data suggest that ingesting the more rapidly absorbed caseinate elicits a greater anabolic stimulus compared with ingesting micellar casein. This insight expands other reviews [22] and the common perception that whey protein, due to amino acid composition (high EAA, BCAA and leucine content) and rapid digestibility properties, is the highest-quality intact protein source popularised in protein supplements. In summary, these data consistently demonstrate that ingestion of whey protein stimulates a greater resting postprandial response of MPS compared to casein protein in young and older adults. Similarly, a direct comparison between “fast” whey protein and “slow” micellar casein protein reveals a superior post-exercise response of MPS to whey protein ingestion in young and older adults.

Variation in the time periods over which MPS or NBAL was measured also may explain the discrepant findings. An interesting observation is that studies reporting a greater response of MPS to whey compared with casein protein conducted measurements of MPS over a 4 h period or less after protein ingestion [15,16], whereas studies reporting no differences between whey and casein conditions obtained measurements of MPS or NBAL over 5 h or more [18,19]. It is conceivable that “rapidly” digested whey protein stimulates a greater response of MPS in the early postprandial period (≤4 h), however this advantageous “muscle protein anabolic response” is cancelled out in the late (≥4 h) postprandial period by the more “slowly” digested casein. Whereas this notion is supported by currently available data, more studies are necessary to substantiate this speculation. Moreover, given the disparate digestive properties and subsequent differences in pattern of blood amino acid appearance between whey and micellar casein protein, physiological rationale underpins the notion that casein should be ingested pre-exercise, whereas whey protein should be ingested post-exercise. However, despite promising rationale [23] surprisingly no study has directly compared the post-exercise response of MPS to ingestion of casein protein before exercise *vs.* whey protein after exercise. Future confirmatory work in young and older adults is necessary to strengthen the quality of this evidence.

Three other direct comparisons of isolated types of intact protein have been studied in young adults: whey *vs.* soy protein which is relatively low in leucine (~7.5% of total protein) content, whey *vs.* rice protein which is slowly digested and relatively low in leucine (~8% of total protein) and casein *vs.* soy protein. A similar resting postprandial response of mixed-MPS to ingestion of whey and soy protein has been reported [15]. However, acute metabolic data that demonstrate a greater post-exercise response of mixed-MPS with whey compared with soy protein ingestion [15] are consistent with a tightly controlled longitudinal endpoint study of ~20 participants [24] that measured greater gains in LBM during a nine-month resistance training period with whey compared to soy protein supplementation. A smaller-scale (*n* = 12 per condition) well-controlled (administration of meal plans) study that compared whey and rice protein isolate supplementation observed similar gains in LBM between conditions during an eight-week training period [25]. Finally, greater rested and post-exercise responses of MPS were reported with soy compared with casein protein ingestion [15]. In summary, given the sparse body of evidence for each comparison (one or two studies), there remains ample scope for future work that compares the response of MPS and SMM to ingestion of various isolated types of intact protein, both from animal (e.g., egg, fish, *etc.*) and plant (e.g., lentil, quinoa, maize, hemp, *etc.*) sources in young and older adults [26].

#### 3.1.2. Protein Blends

A protein blend combines two or more intact proteins. The scientific rationale for ingesting a protein blend is that combining more than one type of protein will capitalise on the unique digestive properties of each type of protein, allowing for an optimal blood availability of amino acids to increase the amplitude and duration of MPS stimulation. The efficacy of a protein blend for the stimulation of MPS was first evaluated by two studies in young adults that compared the ingestion of skimmed milk (casein + whey protein) with isolated soy protein [27,28]. The finding of a greater acute post-exercise response of mixed-MPS and NBAL with milk compared to soy protein ingestion [27] was extended by a longitudinal study that measured a greater increase in LBM after 12 weeks of resistance training in the milk compared to soy protein condition [28]. However, a recent study demonstrated milk ingestion elicits a similar post-exercise response of MPS compared with beef ingestion in young adults [29]. Two other studies compared the post-exercise response of MPS to ingestion of a protein blend (soy + casein + whey protein) with an isolated whey protein control in young adult men [30,31]. The protein blend composition was 25% whey protein, 50% casein and 25% soy protein. Conditions were matched for total EAA (~8.8 g) and leucine (~1.9 g) content, however, the blend condition comprised a greater total protein content compared with the whey protein condition (~19.3 *vs.* ~17.7 g). As anticipated, in both studies [30,31] the amplitude of rise in amino acid concentrations during the early postprandial period was greater in the whey protein compared with protein blend condition. However, with the exception of valine, and to a lesser extent phenylalanine, ingestion of the protein blend failed to sustain elevated plasma amino acid (leucine, isoleucine, total BCAA) concentrations during the late (2–4 h) postprandial period compared with whey protein ingestion. Since the casein source included in the blend was sodium caseinate, which exhibits similar transient amino acid kinetics to whey protein [17,18], it was not surprising that no difference in the duration of increased amino acid availability was observed between protein blend and whey protein conditions. In both studies, the response of mixed [30] and myofibrillar [31] MPS followed the same pattern. At 0–2 and 0–4 h post protein ingestion, a similar increase in the response of MPS above basal values was observed between conditions. These data suggest that whey protein ingestion is similarly effective compared to a dose-matched (for leucine content) protein blend for the stimulation of MPS. Interestingly, despite a similar amino acid profile during late recovery, over the 2–4 h postprandial period, the response of MPS was increased above basal rates in the protein blend condition only. Although these data imply that the duration of MPS stimulation may be extended with a protein blend compared with an isolated type of intact whey protein, this observation also may be an artifact of the additional total protein content of the blend condition compared with the whey protein control. Moreover, the physiological significance of stimulating a greater response of MPS during the late (2–4 h) acute recovery period, without augmenting the aggregate (0–4 h) acute response of MPS, is not obviously apparent. Future work also is warranted to evaluate the response of MPS and SMM to other protein blend combinations, including egg, rice and hemp protein. The implications of these data are of particular relevance to the protein industry that is interested in producing cheaper and more sustainable protein-based products.

An important line of research worthy of future investigation is comparing the response of MPS to animal and plant-derived protein sources, or blends of plant-derived proteins [26]. In particular, combinations of plant-derived protein sources with divergent amino acid profiles that when combined allow for a “complete” EAA profile (e.g., relative to animal-derived proteins, wheat is low in lysine yet high in methionine, whereas lentil is high in lysine, yet low in methionine). A recent study reported a similar increase in SMM with the post-exercise ingestion of pea protein compared with whey protein [32]. However, the limited information available in humans implies that animal-derived protein sources stimulate a greater response of MPS compared with plant-derived protein sources [15,28]. However, the overall completeness, applicability and quality of evidence are weak. To date, a limited number of controlled laboratory studies in humans has directly compared the acute response of MPS to ingesting an animal-derived compared to a plant-derived protein source. No acute metabolic studies in humans have compared other animal-derived protein-rich foods, such as eggs, yoghurts, meat and fish with other plant-derived protein-rich foods, such as lentil, maize, pea, rice and wheat. The implications of these data are particularly relevant to the protein industry for aiding the production of more economically and environmentally sustainable protein-based products [33].

#### 3.1.3. Manipulating Amino Acid Composition

Several studies have investigated the impact of manipulating the composition of an amino acid/protein source for stimulating an increased response of MPS to amino acid/protein ingestion [34,35,36,37]. In terms of amino acid profile, the leucine content of a protein source is of particular importance for stimulating a postprandial response of MPS. Leucine not only provides substrate for the synthesis of new muscle protein, but also serves as a key anabolic signal for skeletal muscle by activating enzymes within the mammalian target of rapamycin (mTOR) signalling pathway [38]. Indeed, the leucine threshold hypothesis [39] has been proposed to explain the observation that young muscle appears relatively sensitive to the anabolic action of small (~1 g) quantities of ingested leucine, whereas older muscle requires ≥2 g of leucine (typically contained in ~20 g of high-quality protein) to increase MPS above resting rates [40]. Accordingly, studies have manipulated amino acid composition in two ways: by adding leucine to an amino acid source or modifying the leucine profile of an AA source. In addition, longitudinal studies have investigated the impact of chronic leucine supplementation on long-term changes in SMM.

Based on available evidence, the efficacy of adding leucine to an amino acid source or modifying the leucine profile of an amino acid source for increasing the stimulation of MPS depends on the interaction of two factors. These factors include the leucine content of the original amino acid source and whether the amino acid source was ingested at rest or after exercise. Two studies in older adults demonstrated the addition of leucine (3.5/2.5 g) to a casein protein (30/20 g) source increased the resting postprandial stimulation of mixed-MPS [39,41]. Conversely, studies in young [42] and older [43] adults reported a similar post-exercise response of mixed-MPS to coingesting leucine (3.4 g) with a whey protein (16.6 g) plus CHO mixture compared to whey protein alone. With regards to modifying leucine profile, studies in young [34] and older [44,45] adults matched the dose of ingested EAA (6.7/10/10 g) between conditions, but manipulated the leucine content (2.8/3.5/3.5 g) of the ingested EAA source. Study outcomes were dependent on the dose of ingested EAA. Leucine-enriched EAA ingestion increased the resting postprandial [34] and post-exercise [44] response of MPS to a suboptimal (for maximal stimulation of MPS—see Amino Acid/Protein dose) dose of EAA, but not to an optimal (for maximal stimulation of MPS) dose of EAA in young [34,45] and [44] older adults. In summary, on the basis of available evidence, leucine coingestion and leucine enrichment effectively stimulates an increased resting postprandial response of MPS to an amino acid source, such as casein protein, that contains a relatively low leucine content (*vs.* whey). In contrast, adding leucine to an amino acid source such as whey protein that already contains sufficient leucine to stimulate a pronounced rise in blood leucine concentration, and thus surpass the leucine threshold for stimulation of MPS, is surplus to increasing post-exercise rates of MPS.

Other studies have manipulated the leucine content of a protein source. A recent study in young adults measured the resting postprandial and post-exercise response of myofibrillar-MPS to ingestion of 25 g whey protein (optimal dose) compared to 6.25 g of whey protein (suboptimal dose) in young adults [46]. Whereas the protein dose was not matched between conditions, leucine intake was equated by adding 2.25 g of leucine (to match the leucine content of the 25 g whey protein dose) to the lower protein dose, thus introducing a leucine-enriched suboptimal dose of whey protein. The impact of leucine-enriching a lower dose of whey protein on the stimulation of MPS differed between resting and post-exercise conditions. In rested muscle, ingestion of a leucine-enriched 6.25 g dose of whey protein resulted in rates of MPS similar to those stimulated with ingestion of a 25 g dose of whey protein. Likewise, ingestion of an EAA-enriched (with the exception of leucine) suboptimal dose of whey protein stimulated a similar MPS response compared with the ingestion of 25 g whey protein. However, notwithstanding the equivalent amount of leucine ingested, an inferior post-exercise response of MPS was observed with ingestion of 6.25 g of leucine-enriched whey protein compared to 25 g of whey protein. This differential response between rested and exercised states may be reconciled by the enhanced ability of muscle to utilise ingested amino acids for the stimulation of MPS following exercise [47]. Hence, it may be speculated that in this study [46], EAA availability was rate limiting for potentiating the post-exercise response of MPS to a suboptimal dose of whey protein. These results support the notion that, rather than blood leucine availability *per se*, the availability of a full complement of EAA is the critical factor for stimulating a maximal response of MPS during exercise recovery.

A follow-up study in young adults by the same authors [35] demonstrated a greater post-exercise response of MPS to ingestion of 25 g of whey protein compared with ingestion of a low dose (6.25 g) of whey protein plus additional leucine (a total of 3 g of leucine) when ingested as part of a mixed macronutrient beverage. However, ingestion of a higher dose of leucine added to 6.25 g of whey protein (totalling 5 g of leucine) resulted in a similar post-exercise response of myofibrillar-MPS to ingestion of 25 g of whey protein. Collectively, these data [35,46] suggest that enriching a suboptimal dose of whey protein with leucine may potentiate the post-exercise response of MPS to a suboptimal protein dose, but only when the suboptimal protein dose is consumed alongside other macronutrients and is leucine-enriched above a certain undetermined threshold.

Based on the rationale that older adults often experience low levels of appetite [48] and routinely consume suboptimal doses of protein, a similar study [49] was recently conducted in older adults. The ingestion of a leucine-enriched (1.2 g) suboptimal dose of EAA (3 g) stimulated a similar resting postprandial and post-exercise response of myofibrillar-MPS compared to a 20 g whey protein bolus containing 9.6 g of EAA and 2 g of leucine. These data suggest that a less satiating (low energy) leucine-enriched suboptimal dose of EAA stimulates a similar resting and post-exercise response of myofibrillar-MPS compared with ingestion of a larger bolus dose of whey protein in older adults. Hence, fortifying a suboptimal quantity of protein with leucine may be a viable strategy for promoting MPS and increasing SMM in older adults. Given that the optimal dose of whey protein to stimulate a maximal post-exercise response of MPS has been shown to exceed 20 g in older adults (see Amino acid/Protein dose), it remains unknown if a leucine-enriched protein source rescues a maximal response of MPS in older adults. Future studies should be designed to provide a similar comparison between a leucine-enriched suboptimal protein dose (*i.e.*, 20 g of whey protein) and an optimal protein dose (~40 g of whey protein) in older adults during exercise recovery.

Finally, two studies in older adults have evaluated the impact of chronic leucine supplementation on outcome measures of SMM and reported equivocal findings [50,51]. Whereas two weeks of leucine supplementation increased the resting postabsorptive and postprandial response of MPS to a suboptimal dose of EAA plus CHO in one study [50], Verhoeven *et al.* [51] reported no change in LBM after 12 weeks of leucine supplementation. Based on these contrasting findings, the efficacy of a prolonged period of leucine supplementation on outcome measures of SMM remains unclear in older adults and warrants investigation in young adults.

#### 3.1.4. Protein Form

Three studies in older adults have manipulated the form of an amino acid/protein source and measured resting postprandial rates of MPS [17,52,53,54]. Koopman *et al.* [52] compared liquid supplements of intact casein and casein hydrolysate and reported a greater blood amino acid availability, and a trend for a greater response of MPS, to ingestion of casein hydrolysate. The same research group recently reported that ingestion of casein in its naturally occurring milk matrix form resulted in a reduced blood amino acid availability (possibly due to delayed amino acid digestion/absorption kinetics), but did not modulate postprandial rates of MPS compared with ingestion of isolated intact micellar casein [53]. A similar result was reported by Pennings *et al.* [54] whereby the ingestion of minced beef, that is easily masticated and digested, stimulated a more rapid increase in arterialised blood EAA availability compared with an equivalent amount of intact steak, however no difference in the 6 h postprandial response of MPS was observed between conditions. These findings [17,53] suggest that, at least in the early resting postprandial period, the rate of blood amino acid availability does not translate into an increased stimulation of MPS. However, it must be recognised that these findings are in the context of a single feeding period under resting conditions. Whether a more rapid blood amino acid availability stimulates a greater response of MPS in the context of repeated feeding and/or during exercise recovery deserves consideration.

### 3.2. Amino Acid/Protein Dose

Several acute metabolic dose-response studies have been designed to characterise the optimum dose of amino acids/protein contained in a single serving for the maximal stimulation of MPS [10,47,55,56,57,58]. These studies examined a range of protein sources, including free crystalline amino acids, intact proteins and complete foods in young and older adults at rest and during exercise recovery.

#### 3.2.1. Young Adults

The optimal dose of ingested amino acids/protein for stimulating a maximal resting postprandial response of MPS is well established in young adults. In the context of a realistic meal-like setting, ingesting a standard portion of lean beef (containing ~30 g protein) was shown to stimulate a similar response of MPS compared with an over-sized portion of lean beef (containing ~90 g protein) [59]. Although a study design that compares only two conditions does not allow for a true dose-response relationship to be characterised, these data suggest a saturable protein dose exists regarding the feeding-induced stimulation of MPS. Consistent with the notion of a saturable dose of protein, we [47] and others [10] observed a plateau in the resting postprandial response of MPS to ingesting 10 g of EAA (2.5 < 5 < 10 = 20 g) [10] or 20 g of intact whey protein (10 < 20 = 40 g) [47]. The ingestion of 20 g EAA [10] or 40 g intact protein [47] failed to elicit an additional resting postprandial stimulation of MPS. Instead, we [47] reported a pronounced stimulation of irreversible amino acid oxidation and ureagenesis, implicating a shift toward fates of ingested amino acids other than MPS. Taken together, these data [10,47] often are interpreted to suggest that, when expressed as an absolute intake, 10 g of EAA (equivalent to ~20 g of protein) is the optimal dose for stimulating a maximal response of MPS in young adults at rest. Expanding these data, a retrospective analysis of previous studies revealed that, expressed relative to body mass, the optimal protein dose for maximal stimulation of MPS in young adults at rest is 0.24 g/kg body mass/serving [60].

In young adults, the optimum dose of protein to ingest during exercise recovery is less well defined. We [47] and others [61] reported no statistical difference in the post-exercise response of MPS to ingestion of 20 compared to 40 g of protein. However, it was intriguing that both studies [47,61] reported an ~10% increase in mean values for the post-exercise stimulation of MPS when the protein dose was increased from 20 to 40 g. Given that increasing the dose of ingested protein from 10 to 20 g stimulated a ~20% greater post-exercise response of MPS without a marked increase in amino acid oxidation or urea production, a diminishing return in terms of stimulating MPS, at the very least, was achieved with ingestion of >20 g of protein [47,61]. The physiological relevance, in terms of long-term changes in SMM, of a 10% increase in the response of MPS during exercise recovery is unknown and warrants further investigation.

#### 3.2.2. Older Adults

In older adults, the optimal dose of ingested protein at rest and during exercise recovery is not well established. Consistent with young adults, Symons *et al.* [59] reported a similar resting postprandial response of MPS to ingesting 113 g (~30 g protein) compared with 340 g (~90 g protein) of lean beef. Moreover, the seminal EAA dose-MPS response study by Cuthbertson and colleagues [10] reported a similar resting stimulation of myofibrillar-MPS with the ingestion of 20 (≈40 g protein) or 40 g (≈80 g protein) of EAA in older adults. Hence, in the context of stimulating a postprandial response of MPS, a saturable dose of ingested protein also exists in older adults. However, several recent dose-response studies of intact protein sources [55,57,58] and protein-rich foods [56] in middle-aged (~60 y) [56] and older adults [55,57,58] failed to observe a saturated response of MPS to graded protein intakes. These studies reported a dose-dependent, graded increase in the response of MPS to increasing doses (0–40 g) of intact whey protein [55,58], soy protein [57] and minced beef [56]. Since no previous study has observed a plateau in the response of MPS to increasing doses of ingested protein [55,56,57,58], the optimal single bolus dose of ingested protein for stimulating a maximal response of MPS in older adults cannot be firmly established.

Despite being inconclusive, two lines of evidence provide an informed estimate of the optimal protein dose for stimulating a maximal response of MPS in older adults. First, previous work has demonstrated that ingesting >36 g of beef protein [56] or 35–40 g of whey protein [55,58] stimulated a pronounced increase in the rate of irreversible amino acid oxidation. These data [55,58] imply the rate of MPS was approaching, or had indeed reached, an upper limit with ingestion of 35–40 g of protein. Second, the maximal effective protein dose at rest is higher in older compared with young adults. A retrospective analysis of previous studies [60] estimated that, when expressed relative to body mass, the dose of protein required to stimulate a maximal response of MPS at rest was ~68% greater in older (0.40 g/kg body mass) *vs.* young (0.24 g/kg body mass) adults. Moving forward, to refine the optimal protein dose for the maximal stimulation of MPS in middle-aged or older adults, future studies should measure the postprandial response of myofibrillar-MPS to 0, 20–40 and 50–60 g doses of ingested protein.

In addition to age, several other nutritional, physiological and/or methodological factors could impact the optimal dose of protein for the maximal postprandial stimulation of MPS in young and older adults. Protein source has been shown to affect the dose-response relationship in older adults. A greater dose of soy protein (≥40 g) [57] was required to stimulate a comparable postprandial MPS response to whey (≥20 g) protein [58]. As such, a rightwards shift in the dose-response relationship was observed with soy protein compared with whey protein. Intuitively, these findings suggest that protein source alters the optimal protein dose for the maximal stimulation of MPS in older adults.

Physiological factors, including body composition and sex-differences, also may impact the dose-response relationship. It is intuitive that individual differences in SMM will affect the optimal protein dose for maximal stimulation of MPS. However, no study has compared the dose-response relationship between individuals with higher *vs.* lower amounts of SMM. Hence, a protein dose exceeding 20 g may be optimal in young adults with high amounts of SMM, particularly during exercise recovery when muscle is sensitised to protein ingestion [8]. Whereas a sex-specific difference in the response of MPS to exercise and nutrition has not been consistently shown in young adults [62,63,64], sexually dimorphic postprandial responses of MPS have been shown in older adults [65]. Thus, although not directly evaluated, these data suggest that sex-specific differences are more likely to affect the optimal single bolus dose of protein in older compared with young adults. Future studies are warranted to test this thesis.

### 3.3. Amino Acid/Protein Timing

The majority of studies have focused on the timing of amino acid/protein ingestion after exercise. Whereas resistance exercise stimulates MPS for at least 48 h during recovery, the magnitude of the post-exercise response of MPS diminishes over time (*i.e.*, 3 > 24 > 48 h) [66]. This time resolution could be explained by the notion that, as time elapses, muscle progressively loses anabolic sensitivity to protein ingestion. An extreme interpretation of this concept is the belief that the anabolic responsiveness of skeletal muscle will be impaired—or even abolished—if an amino acid/protein source is not ingested within as little as 45–60 min following exercise [67]. This time period has been coined the “anabolic window of opportunity.”

The timing of amino acid/protein ingestion before and during exercise also should be considered in the context of stimulating MPS. In theory, amino acid/protein ingestion before and/or during exercise increases blood amino acid concentrations at a time when blood flow also is increased by exercise. During exercise, a net loss of muscle protein is apparent because MPS is either decreased [68] or unchanged [69], whereas MPB is (generally) increased [66]. Moreover, the stimulation of MPS by protein ingestion is refractory, with a latent period of ~1 h [70]. Intuitively, ingestion of an amino acid/protein source before or during exercise, will increase amino acid delivery to skeletal muscle during and immediately post-exercise and counteract the net loss of muscle protein during exercise and in the initial post-exercise recovery period by providing additional substrate for the stimulation of MPS.

Scientific rationale exists also to support the notion that post-exercise amino acid/protein ingestion should be timed in relation to CHO intake. The post-exercise response of NBAL to CHO ingestion is delayed until ~1 h after CHO ingestion [71]. Given that the post-exercise response of NBAL to ingested amino acids is rapid [72], one may speculate that delaying protein ingestion for 1 h after CHO ingestion may superimpose these muscle protein anabolic responses. Thus, it could be argued that amino acid/protein timing should consider the timing of other ingested nutrients, as well as proximity to exercise.

#### 3.3.1. Time-Focused *vs.* Time-Divided Amino Acid/Protein Timing

Surprisingly few studies have compared the impact of time-focused (amino acid/protein ingestion in close temporal proximity to exercise) and time-divided (amino acid/protein ingestion at times other than close to exercise) amino acid/protein ingestion on MPS or SMM. Acute metabolic studies do not support the notion that timing amino acid/protein ingestion immediately post-exercise is critical for optimising the muscle anabolic response. These data reveal a similar response of MPS and NBAL to EAA ingestion 1, 2 or 3 h following resistance exercise in untrained young men [73,74,75]. Hence, it has been argued that the purported “anabolic window of opportunity” may extend beyond the first hour or less following exercise [76]. In addition, a recent study demonstrated protein ingestion 24 h following resistance exercise resulted in a greater response of MPS than protein ingested with no exercise [77]. A direct comparison of the response of MPS to ingestion of protein immediately and 24 h following exercise has yet to be made and thus the stimulation of MPS could, in fact, be slightly greater with protein ingestion immediately following, rather than 24 h after exercise. Nonetheless, it is clear, at least in young adults, that skeletal muscle is still responsive to protein ingestion for at least 24 h following exercise [77]. Thus, according to results from acute metabolic studies, the importance of immediate post-exercise amino acid/protein ingestion does not seem as critical as has often been championed [67,78].

Longitudinal endpoint studies that investigated the efficacy of timing amino acid/protein ingestion in close temporal proximity to exercise for increasing SMM, report inconsistent and, in some cases, puzzling results. A study by Cribb and Hayes [79] reported the ingestion of protein immediately before and after each training session (time-focused protein supplementation regimen) over a 10-week training period resulted in greater improvements in LBM, cross-sectional area of type II muscle fibres and strength compared with ingestion of protein before breakfast and prior to bedtime (time-divided protein supplementation regimen). Similarly, Esmarck *et al.* [80] reported SMM gains after 12 weeks of resistance training in a group of older adults that consumed a protein supplement (within a mixed macronutrient beverage) immediately after a training session, whereas no change in SMM and negligible strength gains were achieved in the group that consumed protein 2 h after exercise. However, it is easy to be sceptical about these data [80]. The magnitude of muscle hypertrophy measured with immediate post-exercise ingestion of the protein supplement was similar to that reported in other resistance training studies with older adult volunteers that included no particular feeding intervention [81,82]. Hence, on closer inspection, the results of this study [80] suggest that immediate post-exercise ingestion of protein does not confer any advantage over resistance training with unsupervised nutrition, at least in older adults. Moreover, it should be noted that waiting 2 h to ingest the protein actually inhibited the “normal” anabolic response to resistance training, making these results [80] puzzling and difficult to interpret. In contrast, other longitudinal studies in young adults fail to support the notion that protein ingestion in close temporal proximity to resistance exercise is critical for maximising SMM. Accordingly, studies by Burk *et al.* [83] and Hoffman *et al.* [84] reported time-focused protein supplementation resulted in a similar [84] or inferior [83] change in LBM after training compared to time-divided protein supplementation. Given that resistance training is an established anabolic stimulus for increasing SMM, it may be considered surprising that no improvement in LBM was observed following the training period with the time-focused supplementation regimen.

#### 3.3.2. Pre- *vs.* Post-Exercise Timing of Protein Ingestion

Other timing considerations may hold similar importance as post-exercise protein timing for optimising the response of MPS. Indeed, ingestion of an EAA plus CHO mixture immediately pre-exercise stimulated a greater response of MPS during 2 h of exercise recovery compared with ingesting an identical EAA-CHO mixture immediately post-exercise [74]. However, an acute study of similar design in young adults, but this time ingesting intact whey protein, reported no difference in NBAL during exercise recovery between pre and post-exercise whey protein conditions [85]. Moreover, the exercise-induced stimulation of MPS was similar when a protein-containing meal was ingested 2 h prior to exercise [86] compared with when an amino acid source was provided after exercise [9,87]. Accordingly, a longitudinal endpoint study reported similar increases in LBM after 12 weeks of resistance training between groups of older adults that consumed a protein blend supplement either before or after each exercise session [88]. Taken together, these data [9,86,87,88] suggest that skeletal muscle is, at the very least, comparatively responsive to amino acid/protein ingested pre or post-exercise.

#### 3.3.3. Timing of Amino Acid/Protein Ingestion in Relation to Other Nutrients

Only one study has tested the hypothesis that separating, rather than combining, the post-exercise ingestion of amino acids and CHO increases the muscle anabolic response during exercise recovery [75]. However, despite the separate ingestion of EAA and CHO stimulating a transient physiological increase in NBAL in the first 2 h of recovery, no difference in NBAL was demonstrated between combined or separate ingestion of EAA and CHO over an extended 6 h recovery period [75]. Thus, from a practical perspective, separating ingestion of EAA and CHO should be considered unlikely to be an important component of protein recommendations for maximising the muscle protein anabolic response during exercise recovery. Instead, a more simple approach of ingesting CHO and EAA together is sufficient to engender increased muscle anabolism.

#### 3.3.4. Bedtime Protein Feeding

The timed ingestion of amino acids/protein in relation to overnight recovery is a topic of recent investigation [89,90]. It has been proposed that ingesting a protein source that releases amino acids slowly into the blood immediately prior to sleep promotes a more positive NBAL during overnight recovery [89,91]. By maintaining increased blood amino acid availability throughout the night, it may be possible to stimulate MPS and/or attenuate MPB, thereby improving NBAL during overnight recovery from exercise—a period often associated with an extended phase of negative NBAL. Indeed, the timed ingestion of protein before bedtime has been shown to increase the nighttime stimulation of MPS in young and older adults [89,91], and thus may be an effective strategy to increase muscle anabolism during overnight recovery. However, in previous studies [89,90], no time control condition was included, e.g., protein ingestion at a time point other than before bedtime. Hence, the impact of protein timing *per se* cannot be distinguished from the increased protein intake over the day.

### 3.4. Amino Acid/Protein Pattern

Amino acid/protein pattern accounts for the dose, timing and frequency of ingestion. A balanced pattern is characterised by the equal spread of total daily protein intake between servings, whereas, an unbalanced pattern—shown to be the norm for young [92] and older [93] adults—is characterised by consuming a large proportion of total daily protein intake in a single serving, usually in the evening meal. The aggregate daytime response of MPS is a direct function of the cumulative MPS response to each individual protein serving during the course of a day. In theory, the divergent profiles of blood amino acid concentrations associated with manipulating the timing and frequency of amino acid/protein intake during the course of a day will explain differences in the cumulative response of MPS to balanced and unbalanced protein meal patterns. Accordingly, acute metabolic studies have investigated the influence of amino acid/protein feeding pattern on the aggregate daytime stimulation of MPS while longitudinal endpoint studies have investigated the influence of protein meal pattern on chronic changes in SMM and strength.

#### 3.4.1. Young Adults

Four studies in young adults have investigated the influence of protein pattern on the daytime stimulation of MPS or chronic changes in SMM [94,95,96,97]. Acute metabolic studies are not comparable given the discrepancies in research design including exercise state (rest *vs.* post-exercise), and protein feeding regimen (intact protein *vs.* mixed macronutrient meals). Moreover, the unbalanced pattern implemented in these study designs may be considered somewhat extreme and not reflective of real-world practice. These studies provide ~70% of total daily protein intake in the evening meal [96] which is more than typically consumed during dinner under free-living conditions. Areta *et al.* [94] demonstrated a greater 12 h post-exercise response of myofibrillar-MPS to distributing 80 g of whey protein as 4 × 20 g servings compared with 2 × 40 g servings 6 h apart, or 8 × 10 g servings 1.5 h apart. In a more practical study design, Mamerow *et al.* [96] demonstrated a greater 24 h resting postprandial response of MPS to a balanced meal pattern that distributed 90 g of protein evenly between three meals (3 × 30 g), spaced 3.5–4 h apart *vs.* a conventional [92,93] unbalanced protein meal pattern that biased 70% of daily protein intake towards the evening meal. Hence, despite an equal total daily protein intake (90 g) between conditions, the aggregate daytime stimulation of MPS was greater with a balanced compared to unbalanced protein feeding pattern. A theoretical explanation for the improved aggregate daytime stimulation of MPS with a balanced protein meal pattern may be attributed to the muscle full effect [98] and thus repeatedly reaching the leucine threshold for the maximal acute stimulation of MPS. However, these data are not supported by a recent short-term acute metabolic study [97] that demonstrated no difference in the 3 h resting response of MPS to ingestion of 15 g of EAA either as a single bolus or distributed between four small boluses. Moreover, the only published chronic study by Arnal and colleagues [95] reported no changes in LBM following 14 days of either a balanced or unbalanced protein meal pattern. However, a drawback of this study [95] was that 2/4 meals contained 13–15 g of protein, rather than the optimal 20 g dose [47,61]. At this juncture, acute [96,97] and chronic studies [95] in young adults investigating the influence of protein pattern on MPS and SMM provide inconsistent results. Future studies in young adults should be designed to compare a balanced *vs.* unbalanced distribution pattern of daily protein intake on the daytime stimulation of MPS (under resting and post-exercise conditions) and training-induced changes in SMM, whilst taking into consideration the established optimal dose of protein contained in a single serving for young adults.

#### 3.4.2. Older Adults

Two studies have investigated the influence of protein meal pattern on the response of MPS and SMM in older adults [99,100]. In contrast to studies in young adults, no study has reported that protein meal pattern affects the aggregate response of MPS to total daily protein intake. Kim and colleagues [100] reported no difference in the 22 h response of MPS to an unbalanced pattern that biased 65% of daily protein intake towards the evening meal compared with a balanced pattern that spread total daily protein intake evenly between meals. In this study [100], the balanced pattern consisted of three meals that each contained a protein dose (~37 g) that was likely sufficient for stimulating a maximal resting postprandial response of MPS in older adults [55,58,100]. However, the statistical power of this dataset [100] may be considered to be insufficient given that the sample size of the unbalanced group was only four participants. The only published chronic study by Arnal and colleagues [99] reported no changes in LBM following 14 days of either a balanced or unbalanced protein meal pattern. Thus, on the basis of statistical analysis, results are consistent between acute [100] and chronic [99] studies that investigate the influence of protein pattern on MPS and SMM. To date, no study has investigated the influence of protein feeding pattern on the aggregate post-exercise response of MPS to daily protein intake in older adults.

### 3.5. Macronutrient Coingestion

Irrespective of whether protein is consumed in food (mixed-macronutrient meal) or supplement (liquid beverage or solid bar) form, it is often coingested with CHO and/or fat. Hence, it is important to understand the impact of macronutrient coingestion on MPS and SMM.

#### 3.5.1. Carbohydrate Coingestion

Macronutrient coingestion alters physiological factors known to regulate the stimulation of MPS. CHO coingestion increases plasma insulin concentrations compared to CHO [101] or protein [102] alone and the anabolic action of insulin on muscle protein metabolism is two-fold. First, under conditions of sufficient amino acid availability [103,104], insulin increases amino acid delivery to skeletal muscle (a rate limiting step in the stimulation of MPS) by increasing capillary recruitment and microvascular perfusion [105]. Second, insulin initiates a suppression of MPB via the ubiquitous proteasome pathway [106]. Therefore, CHO coingestion theoretically has the potential to facilitate the stimulation of MPS and suppress the stimulation of MPB.

A systematic series of hypothesis-driven studies has investigated the influence of CHO coingestion on the response of muscle anabolic response to an amino acid/protein source. Based on available evidence, the efficacy of CHO coingestion to increase the muscle anabolic response and SMM in response to amino acid/protein ingestion is dependent, at least in young adults, on the dose of ingested amino acids/protein. Two acute metabolic studies indicate that coingesting CHO with ~6 g of amino acids increased the muscle protein anabolic response in young adults, compared with the independent ingestion of amino acids [107,108]. These findings of a 60% greater utilisation of ingested amino acids [108] and suppression of urinary 3-MH excretion [107]—a crude marker of MPB—in response to exercise with CHO-amino acid coingestion indicate a greater acute stimulation of MPS and inhibition of myofibrillar-MPB, respectively. Accordingly, the findings of Bird *et al.* [107] were extended to a longitudinal training study [109] whereby young adults achieved greater gains in type II muscle fibre cross-sectional area after 12 weeks of resistance training when consuming a CHO plus amino acid-containing supplement during each exercise session compared with an amino acid-only supplement. As detailed previously, in the absence of sufficient blood amino acid availability [9], the anabolic action of a CHO-mediated increase in blood insulin concentration is likely to target a suppression of MPB, rather than stimulation of MPS [3]. Prior work demonstrated the insulin-mediated suppression of MPB to be linearly graded up to an insulin concentration of ~30 uU/mL [106]. Taken together, these data in young adults suggest the increased muscle anabolic response to coingesting CHO with small (≤6 g) doses of EAA is mediated by a suppressed response of MPB [106,107,109]. To date, no study has investigated the impact of coingesting CHO with a suboptimal dose of protein (rather than amino acids) on MPS in young or older adults.

A handful of acute metabolic studies in young [3,102,110,111] and older [110,112] adults report that coingesting CHO with a moderate/large dose of amino acid/protein elicits no change in rested [102,110,112] or post-exercise rates of MPS [3,102,111] or MPB [102]. Consistent with these data [3,102,110,111,112], similar improvements in LBM, fibre-specific muscle hypertrophy and strength were reported when resistance-trained young males consumed either a protein or mixed protein-CHO supplement immediately after each exercise bout of a 10 weeks resistance-training period [79]. This absence of an additive effect of protein and CHO was evident despite CHO coingestion stimulating a robust increase in circulating insulin concentrations [102,111]. Given that basal insulin concentrations are known to be sufficient for stimulating MPS in the presence of amino acids [106], the insulin response to moderate or large protein doses could be considered sufficient to saturate mTORC1 signalling, thus rendering the CHO-mediated increase in insulin concentration permissive for increasing the stimulation of MPS.

#### 3.5.2. Fat Coingestion

Preliminary, albeit inconsistent, evidence also suggests that fat coingestion increases the muscle anabolic response [113,114,115]. Mechanistic studies have demonstrated that increasing free fatty acid concentrations in blood had no impact on the responsiveness of NBAL to amino acid ingestion [114,115]. Moreover, results from a recent study demonstrated that coingesting milk fat with casein protein failed to increase the postprandial stimulation of MPS in older adults [53]. In contrast, a study of greater physiological relevance by Elliot *et al.* [113] demonstrated that ingestion of whole-fat milk stimulated a superior post-exercise utilisation of ingested amino acid compared with ingestion of skimmed-fat milk matched for volume (239 g) and similar in protein content (8.0 *vs.* 8.8 g, respectively). To date, no study has directly assessed the response of MPS to coingesting fat with an amino acid/protein source under rested or exercised conditions in young or older adults.

A topic of recent interest is the role of fish oil derived long chain *n*-3 polyunsaturated fatty acids (LC *n*-3 PUFA) in increasing MPS and SMM [116,117,118,119]. Studies in young and middle-aged [119] or older [118] adults have demonstrated that eight weeks of LC *n*-3 PUFA supplementation increased MPS rates, and the phosphorylation status of signalling proteins (mTORC1-p70S6k1 signalling) known to regulate MPS, in response to the intravenous infusion of combined amino acids and insulin. Irrespective of age, no change in basal MPS was observed with LC *n*-3 PUFA supplementation [118,119]. These data [118,119] suggest that, rather than exerting a direct anabolic effect on muscle protein, LC *n*-3 PUFA sensitise skeletal muscle to potent anabolic stimuli, such as amino acids and insulin. Moreover, a prolonged period of supplementation with LC *n*-3 PUFA was shown to enhance muscle mass and function at rest [117] and resistance training-induced improvements in muscle strength and functional capacity in older adults [116]. However, in this study [116], no measurements of SMM were collected and therefore the impact of LC *n*-3 PUFA supplementation, in combination with exercise training, on chronic changes in SMM remains unknown.

Two causal mechanisms are proposed to underpin the anabolic action of LC *n*-3 PUFA. First, LC *n*-3 PUFA may exhibit intrinsic muscle protein anabolic properties by modifying the lipid profile of the muscle phospholipid membrane [118,119]. These structural changes in membrane properties may activate membrane-bound anabolic signalling proteins, such as focal adhesion kinase (FAK) and the downstream anabolic target proteins, protein kinase B (PKB) and mechanistic target of rapamycin (mTORC1) [120]. Secondly, the potential anabolic action of LC *n*-3 PUFA supplementation also may be related to a modulated inflammatory response [121]. The next logical step for this new research topic is to investigate the role of LC *n*-3 PUFA supplementation in sensitising skeletal muscle to more physiologically relevant anabolic stimuli, such as resistance exercise and protein feeding in young and older adults.

## 4. Conclusions and Future Perspectives

Protein guidelines for increasing or preserving SMM are more complex than simply recommending a total daily amount of protein. We have identified several factors involved in protein nutrition, including the source, dose, timing, pattern and coingestion of other nutrients that independently, concurrently and additively influence MPS under resting and post-exercise conditions. Consequently, understanding the interaction between these aforementioned factors of protein nutrition and MPS is critical for contextualising protein recommendations for increasing or preserving SMM in healthy young and older adults.

### 4.1. Implications for Practice

On the basis of published literature collated in this review, we propose the following evidence-based implications for practice.

- Protein guidelines should be customised to the population (young or older adults) and situation (resting or post-exercise condition) of interest. For example, (a) the optimal dose of protein for maximal stimulation of MPS during exercise recovery is greater for older compared to young adults and (b) whey protein has been shown to stimulate a greater response of MPS compared with soy protein during exercise recovery, but not at rest.
- Chronic periods of leucine supplementation will not necessarily facilitate long-term improvements in SMM, given that a full complement of EAA is critical for stimulating a maximal and sustained response of MPS.
- Manipulating the leucine content of a protein source that lacks quality (*i.e.*, the protein source constitutes a low leucine composition) and/or quantity (*i.e.*, an insufficient protein dose for the maximal stimulation of MPS) effectively rescues a submaximal resting postprandial stimulation of MPS. This phenomenon has particular implications for older adults or other populations that often experience difficulties in consuming a sufficiently large dose of protein in each meal serving to stimulate a maximal response of MPS.
- Timing protein intake in close temporal proximity to exercise is recommended, although not critical, for stimulating a maximal response of MPS.
- Coingesting CHO with a suboptimal dose of amino acids/protein may be an effective strategy for “rescuing” a submaximal response of MPS associated with a suboptimal dose of amino acids/protein. However, no additional benefit is gained from adding CHO to a dose of amino acids/protein known to saturate the response of MPS.
- Any beneficial impact of fat coingestion on MPS is likely mediated by the anabolic action of the LC *n*-3 PUFA.

### 4.2. Implications for Research

Table 1 extracts from the main body of text a multitude of future academic research directions in the field of protein nutrition. This grid has been designed to illustrate the independent or interactive effects of the several factors of protein nutrition on the stimulation of muscle protein synthesis. The placement of each question is dependent on the factor of protein nutrition addressed by the question. For example, the question “Can plant-based protein sources stimulate a similar response of MPS compared with animal-based protein sources?” relates to the independent impact of *protein source* on MPS and thus fits in the protein source-protein source space. The question, “What impact does coingesting CHO with a suboptimal dose of protein have on the stimulation of MPS in young and older adults?” relates to the interactive effect of *protein dose* and *macronutrient coingestion* on MPS and thus fits in the protein dose-macronutrient coingestion space. As a general point, current protein recommendations are primarily informed by research designs whereby protein beverages are administered commonly as an isolated protein source. By characterising the response of MPS to the single and multiple bolus ingestion of mixed-macronutrient meals or supplements, it will be possible to tailor more practical and personalised nutrition advice regarding what foods/supplements should be consumed, how much of a food/supplement should be consumed and when food/supplements should be consumed on both rest and exercise training days.

In terms of future perspectives, from a methodological standpoint the field is entering an exciting period to study the role of protein nutrition in modulating muscle protein metabolism [122]. Specifically, a recently validated oral deuterium oxide isotope tracer protocol allows for the relatively non-invasive measurement of free-living, integrated rates of MPS over an intermediate time period (e.g., 1–14 days) [123,124] that, in the future, should be extended to longer time periods [125]. Hence, quantifying fraction-specific rates of MPS to represent skeletal muscle protein remodelling in response to perturbations such as resistance exercise and protein ingestion is possible over acute, intermediate and potentially chronic time periods. Such tools will inevitably expand our existing knowledge regarding protein considerations for optimising SMM in both healthy young and older adults.

As a closing remark, there are a distinct lack of data in females and middle-aged (40–55 years old) adults. Since sex-differences in the response of MPS to feeding have been reported [63,65], future studies should investigate the impact of protein feeding on MPS and SMM in cohorts of female volunteers.

## Figures and Tables

**Table 1:** Proposed future research directions to promote understanding of how several factors of protein nutrition interact to impact the stimulation of muscle protein synthesis (MPS) at rest and during exercise recovery in young and older adults.

	Source	Dose	Timing	Pattern	Coingestion
Source	Can plant protein sources stimulate a similar response of MPS compared to animal protein sources in young and older adults? Do liquid-based forms of ingested protein stimulate a greater response of MPS compared to solid-based forms of protein foods?		What impact does protein source have on the optimal timing of protein ingestion in young adults?	What impact does protein source have on the optimal protein meal pattern for the daytime stimulation of MPS in young and older adults?	
Dose	What impact does protein source have on the optimal protein dose for stimulation of MPS in young adults?	What is the maximal effective dose of protein for the stimulation of MPS in older adults? What influence does individual lean body mass have on the optimal protein dose for stimulation of MPS?			What impact does macronutrient coingestion have on the optimal protein dose for stimulation of MPS in young adults?
Timing	How does the response of MPS during exercise recovery compare between the pre-exercise ingestion of casein *vs.* the post-exercise ingestion of whey protein?		Does the overnight stimulation of MPS with bedtime protein feeding translate into long-term gains in skeletal muscle mass?		What impact does macronutrient coingestion have on the optimal protein timing for stimulation of MPS in young and older adults?
Pattern		What impact does protein dose have on the optimal pattern of protein feeding for the aggregate daytime stimulation of MPS?		What is the impact of protein feeding pattern, combined with exercise, on the aggregate daytime stimulation of MPS in older adults?	
Coingestion		What impact does coingesting carbohydrate with a suboptimal dose of protein have on MPS in young and older adults?		Does the ingestion of protein within mixed macronutrient meals impact the optimal protein meal pattern for the daytime stimulation of MPS?	What is the impact of long chain *n-3* polyunsaturated fatty acid supplementation on the response of MPS to exercise and protein feeding in young and older adults?

## Abbreviations

The following abbreviations are used in this manuscript:

BCAA: branched chain amino acids
EAA: Essential amino acids
LBM: lean body mass
MPS: muscle protein synthesis
MPB: muscle protein breakdown
NBAL: net muscle protein balance
SMM: skeletal muscle mass
